# Trauma Care Training in Vietnam: Narrative Scoping Review

**DOI:** 10.2196/34369

**Published:** 2022-01-24

**Authors:** Ba Tuan Nguyen, Toi Lam Phung, Thi Hong Hanh Khuc, Van Anh Thi Nguyen, Christopher Leigh Blizzard, Andrew Palmer, Huu Tu Nguyen, Thang Cong Quyet, Mark Nelson

**Affiliations:** 1 Menzies Institute for Medical Research University of Tasmania Hobart Australia; 2 Health Strategy and Policy Institute Ministry of Health Hanoi Vietnam; 3 Hanoi Medical University Hanoi Vietnam

**Keywords:** trauma training, Vietnamese medical education system, medical curricula, short course

## Abstract

**Background:**

The model of trauma in Vietnam has changed significantly over the last decade and requires reforming medical education to deal with new circumstances. Our aim is to evaluate this transition regarding the new target by analyzing trauma and the medical training system as a whole.

**Objective:**

This study aimed to establish if medical training in the developing country of Vietnam has adapted to the new disease pattern of road trauma emerging in its economy.

**Methods:**

A review was performed of Vietnamese medical school, Ministry of Health, and Ministry of Education and Training literature on trauma education. The review process and final review paper were prepared following the guidelines on scoping reviews and using the PRISMA (Preferred Reporting Items for Systematic Reviews and Meta-Analyses) flowchart.

**Results:**

The current trauma training at the undergraduate level is minimal and involves less than 5% of the total credit. At the postgraduate level, only the specialties of surgery and anesthesia have a significant and increasing trauma training component ranging from 8% to 22% in the content. Trauma training, which focuses on practical skills, accounts for 31% and 32% of the training time of orientation courses for young doctors in “basic surgery” and “basic anesthesia,” respectively. Other relevant short course trainings, such as continuing medical education, in trauma are available, but they vary in topics, facilitators, participants, and formats.

**Conclusions:**

Medical training in Vietnam has not adapted to the new emerging disease pattern of road trauma. In the interim, the implementation of short courses, such as basic trauma life support and primary trauma care, can be considered as an appropriate method to compensate for the insufficient competency-related trauma care among health care workers while waiting for the effectiveness of medical training reformation.

## Introduction

**Figure 1:**
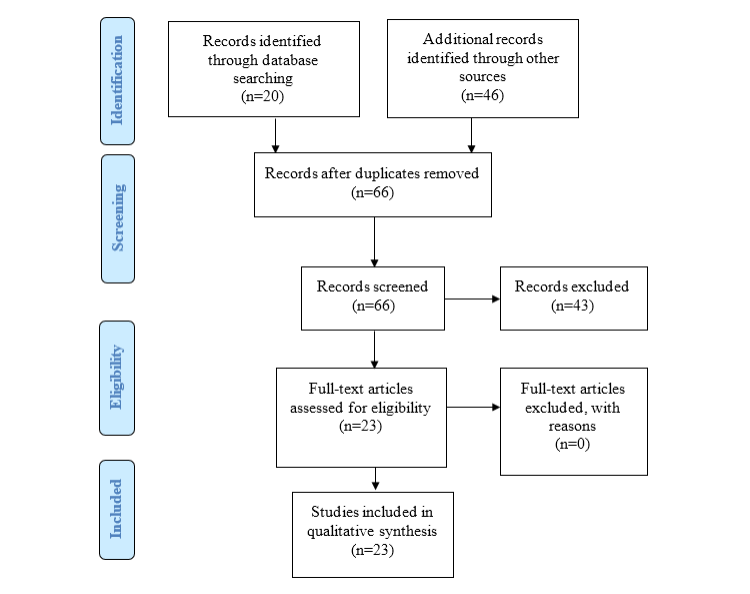
The Preferred Reporting Items for Systematic Reviews and Meta-Analyses (PRISMA) flowchart.

Over the last 2 decades, there has been a sharp increase in private vehicle use in Vietnam consequential to economic and transport infrastructure development. From 2006 to 2018, the number of cars and motorcycles increased by 413% and 317%, respectively, to more than 3.9 and 58 million, respectively [1,2]. Consequently, the number of road accidents has risen in parallel with the number of cases, increasing from approximately 11,000 in 2009 to a peak of 43,000 in 2011 [1]. The latest figure (1st quarter of 2021) witnessed an improvement but was still an alarming number with 3206 cases involving around 1672 deaths and 2386 injuries [3]. Due to the prevalence of motorcycle transport, not surprisingly, head injuries accounted for more than three-quarters (79%) of road trauma injury (RTI) cases presenting to the emergency department, followed by multitrauma (9.2%) and limb injuries (2.3%) [4]. Trauma is always on the list of the leading causes of mortality and morbidity in all age groups and worldwide [5]. Despite the enforcement of wearing helmets on motorcycles in 2007 [6] and legislation adjustment for drunk driving in 2020 [7], along with HIV/AIDS, RTI is a health burden in Vietnam [3,8,9]. This is because, like other low- and middle-income countries (LMICs), the prehospital care system in Vietnam is underdeveloped. Consequently, 42% of RTI victims died before reaching a health care facility compared with 29% in hospitals [10].

In the Vietnamese health care setting, trauma victims will generally be taken to the grassroot-level center, such as a district or provincial hospital, for initial treatment. However, the health workforce, especially physicians in these settings, mainly consists of general practitioners and nurses who are varied in their level of trauma training. Only 1.7% of health staff in these facilities have specialty training (including trauma specialists) [11]. “Trauma doctors,” as they are referred to in Vietnam, are specialized in trauma care and have been trained in a postgraduate program. They tend to be located in large central health care centers rather than in rural or provincial areas [12]. Consequently, serious trauma patients need to be transferred from commune health stations and district hospitals to a higher-level care facility where there are doctors specialized in trauma care. It has been estimated that 55% of trauma victims present to provincial hospitals, so it can be seen that these centers need trauma training at least to the level of stabilizing patients before transfer [4].

Since the reunification of the country in 1975, the medical education system has been community-based to address a shortage of human resources in Vietnam and to distribute them more equitably. This model, however, has restricted the time exposed to specialist training, including training at both undergraduate and postgraduate levels [13,14]. Nevertheless, medical training, in general, must be adapted to the epidemiologic transition of an emerging economy in which road trauma is a major burden for the health care system. A review in 2012 by Fan et al documented medical training in Vietnam to that date [13], but information regarding the content of training concerning the burden of disease was not well ascertained. Since then, there have been changes in regulations and curricula in most medical universities. This article aims to review how trauma care training is provided in Vietnam and to explore the pattern of such training programs and courses.

## Methods

### Study Design

This was a narrative scoping review [15]. Since there is a lack of a review on trauma care training in Vietnam and the scope of the research question is broad, a scoping review combined with narrative synthesis was deemed to be the most appropriate method [15]. The scoping review design allows us to identify broad and diverse types of trauma care training. A narrative synthesis provides a context in which to describe what is deficient and necessary to address for different participants (ie, trainees). Information for this study was identified, extracted, and charted from various sources, including international and domestically published studies, as well as the gray literature. The review process and final review paper were prepared following the guidelines on scoping reviews [16] and the Preferred Reporting Items for Systematic Reviews and Meta-Analyses (PRISMA) statement [17].

### Eligibility Criteria

Eligible articles/reports for this study were publications that reviewed or reported information concerning the trauma care training in Vietnam. As we also analyzed the content of trauma care training in medical training, the curriculum from medical universities was also included. Papers that reported trauma care training for non-Vietnamese health care workers, not working in the Vietnamese health care system (eg, training for US medics and nurses during the Vietnam War) were excluded.

### Information Sources

We conducted a systematic search for peer-reviewed articles in the English language, which were indexed in the MEDLINE database (through PubMed) in February 2021. We also searched domestic literature through electronic databases at Hanoi Medical University (HMU) and Vietnamese Joint Medical Library [18]. Furthermore, gray literature was identified through an online search via Google Scholar and the websites of major medical universities in Vietnam, Vietnamese Government agencies, and nongovernment organizations.

### Search

The search strategy in PubMed was developed to include studies with the following terms in the title/abstract: “Vietnam” OR “Viet Nam” AND “trauma” together with “training” OR “education” OR “continued medical education” OR “CME.” The details of the search strategy are provided in Multimedia Appendix 1. Meanwhile, the search in the aforementioned Vietnamese sources was performed with equivalent keywords in the Vietnamese language such as “
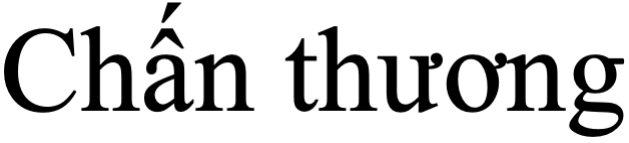
 (trauma)” and “
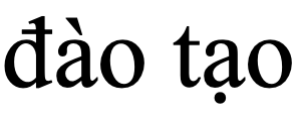
 (training).”

### Selection of Sources of Evidence

Two reviewers (BTN and TLP) independently selected studies. Any discrepancy in the selection was solved by a discussion with a third reviewer.

### Data Charting Process

Key findings from documents in the Vietnamese language were summarized and translated into English. Two reviewers (BTN and THHK) independently extracted data from the included studies and reports using a standardized data extraction form. The extracted information included the year of publication, type of training (undergraduate, postgraduate, and continuing medical education [CME]), number of credits, duration of training, etc. Any disagreement in data extraction between the 2 reviewers was resolved by discussion with a third-party reviewer (TLP, VATN, and HTN).

### Synthesis of the Results

The results were narratively summarized. We further analyzed the curriculum of the undergraduate and postgraduate programs of HMU, as an example, to illustrate the duration of trauma training. The selection of HMU as the showcase is justifiable since HMU is one of the top medical universities in the country and has been assigned by the Ministry of Health (MOH) to develop the training outputs standard for general doctors.

## Results

### Search Results

Our search strategy identified 2 English language articles/reports and 2 Vietnamese language research articles/reports that met the eligibility criteria. We also included 19 gray literature documents (Figure 1). Our literature search did not identify any review articles.

### Undergraduate Training

In 2021, there were 27 medical universities in Vietnam, with the public sector dominating the private and with most of the sites located in urban areas [19,20]. For undergraduate training, the following 2 health care worker training programs currently exist in Vietnam: a 6-year program (doctor training) and a 4-year program (nurse and medical technician training). Each medical university built its curriculum based on the framework curriculum, which was developed and approved by the MOH and the Ministry of Education and Training. The university must have received approval from the MOH before delivering its curriculum [21,22]. This research only describes the curriculum of the 6-year program (general doctor training program).

**Figure 2:**
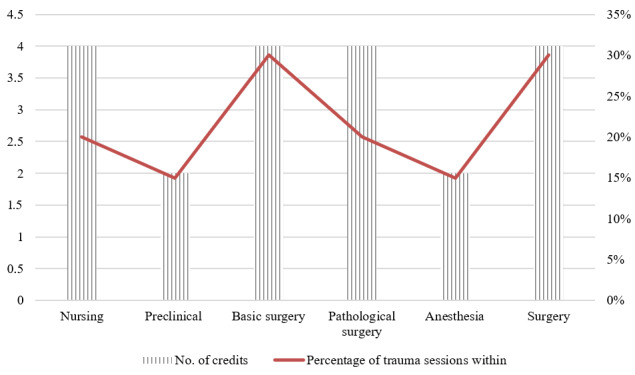
Trauma-related lessons by subject in the curriculum of Hanoi Medical University.

The undergraduate curriculum consists of about 60 subjects, which can be combined into the following 3 categories: basic science, preclinical, and clinical. The curriculum structure is almost identical in all the medical universities and has not changed much in recent years [13]. However, the trauma training theme only accounts for a few lessons in the following 6 subjects: nursing, preclinical, anesthesia, basic surgery, pathological surgery, and surgery (Figure 2).

Trauma lectures appear from the second to the final year of the medical training program (Figure 2). Clinical case testing occurs when students complete their hospital rotation, and then, students are assessed for theory by a multiple-choice examination. Every medical student must complete both theory and practice components with 233 credits in total (each credit is equivalent to 15 lessons, and each lesson is 45 minutes of teaching) during the 6 years at the university to become a doctor [23]. Of these, only about 10 credits have content relating to trauma or first aid training (under 5%). The content of the lessons is varied with more than 40 different tasks such as skills of surgical abdominal examination, clavicle and femur immobilization skills, and skills of examination and first aid for abdominal injuries. To evaluate the academic results, each student takes a clinical-based assessment and multiple-choice question (MCQ) theoretical examination on a computer. In the final year, the content on surgery relating to trauma care is separated into small parts in 10 lessons.

Many students return to local health centers to start working at a commune or at district health facilities right after graduation. First aid and trauma care skills taught at the undergraduate level, therefore, are extremely important for them. However, the current medical training program does not focus on preferential or targeted first aid training for this group of students.

### Postgraduate Training

After completing 6 years of study at a medical university, students have the following 2 main choices: participate in postgraduate training programs immediately (residency training) or practice at the hospital for 18 to 24 months to gain experience and receive a medical practicing license. The license is a prerequisite of some postgraduate training programs (specialized level I [SL-I] and master’s degrees). There are the following types of postgraduate training in Vietnam: clinical training (practice doctor, SL-I, and specialized level II [SL-II]), academic training (master and PhD), and residency training. In all types, the trainees will choose a specific specialized training program (internal medicine, surgery, etc). These forms of training differ in training duration, entry criteria, and output standards. Specifically, the SL-I, SL-II, and master training programs take 2 years, while this duration is 3 years for residency training and from 3 to 7 years for a PhD program. Students have to satisfy the criteria of having 2 to 3 years of experience in the applied specialty and to pass an entrance exam to be eligible for the postgraduate training, except PhD candidates who have distinct requirements. To complete the programs, most of the trainees have to defend their thesis prior to graduation, which will be assessed internally by professors [24], except the SL-I program, for which the trainees only have to pass the final test that normally involves MCQ and clinical-based assessments.

As for residency training, students need to finish both dissertation and graduation examinations [25,26]. The residency training in Vietnam involves both the academic and clinical sectors. As a result, after completing this program, trainees can gain both a master’s degree and residency certification. With these qualifications, graduated resident doctors may be eligible for higher education enrolment in either an academic (PhD) or clinical field (SL-II).

The curricula of all training types follow a unified system that is developed by the MOH. The training content includes basic science subjects, basic medicine groups, foreign languages, and specialty subjects. The duration of subjects is the same across majors, except for specialty subjects. Moreover, there are 27 different medical majors in Vietnam, but no separate major specialized in trauma.

**Figure 3:**
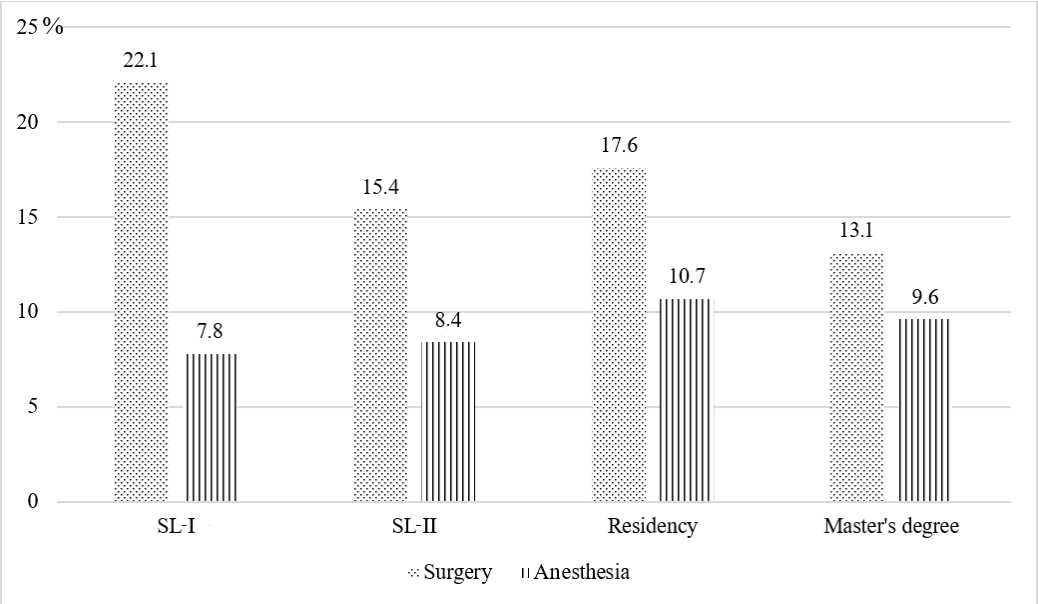
Percentage of lessons related to trauma in 2 postgraduate programs. SL-I: specialized level I; SL-II: specialized level II.

Like the undergraduate program, there is no trauma specialist training in these upper levels, with 2 postgraduate programs that are partially related to trauma care including surgery and anesthesia. However, by analyzing the curricula of these programs, the proportion of formal sessions directly related to trauma care ranged from 13% to 22%. The figure for the latter was even lower at approximately 8% to 11% (Figure 3). The first aid and trauma lessons included such topics as airway management, polytrauma management, and brain trauma resuscitation. Lessons may be theoretical, practical, or both. Of those with both components, practice dominated theory. Unlike the other training streams, doctoral training does not require any course component [27-33]. Most of the theory content is taught to students face to face in class, while the teaching method for the practical component varies from skill stations and clinical scenarios to “bedside learning” in real patients.

### CME, Orientation, and Short-Course Training

#### Orientation or Basic Course

After graduation from medical school, generalist doctors can choose to go directly to clinical practice in primary health care or pursue an orientation course with a duration of 6 to 11 months in a specialty such as surgery, emergency medicine, or internal medicine. There is no entrance examination for the orientation course. This course is a requirement in addition to evidence of 18 months of hospital practice for medical license approval (specialist).

During their training time, trainees need to pass MCQ and clinical-based assessments. After completing the course, they qualify as a “practice doctor.” However, this type of training has been terminated by the MOH since 2019 [34]. After this date, all specialties have created another “basic course.” These courses have a similar structure of training to other specialized “orientation courses.” Of those, the 2 majors most relevant to trauma training are “basic surgery” and “basic anesthesia,” with 31% and 32% trauma training content, respectively [35,36]. Both courses emphasize on practice rather than theory only, as the former dominates the latter regarding training time [35,36]. The 2 programs are considered as the first step of the specialty training process, so they have been designed to equip participants with basic rather than advanced skills. As a result, after graduation, a program participant is likely to be adequately trained at the grassroots level in the health care system.

#### CME and Short-Course Training

Like other countries, participation in CME and short-course training in Vietnam is mandatory for doctors to be able to register and maintain their medical license. The CME and short-course training could be organized by a medical school, hospital, or specialized medical association such as the Vietnamese Association of Traumatology and Orthopedics. Therefore, these training courses are varied in their topics, durations, trainers, and trainees.

The main purpose of these courses is to provide front-line health workers with knowledge and skills to deliver emergency medical care with only basic equipment and facilities. The format of courses is varied (1- to 3-day courses). The number of participants is also different between courses [37,38]. Most participants are doctors (surgeons, general practitioners, and anesthetists) and nurses, but there are some paramedic staff.

Some courses have received very positive feedback from participants. One hundred and eighty medical staff participated in Luong’s study about capacity building [37]. Over 90% of them assessed the training program as suitable between the objectives with the theoretical and practical content. These courses are truly global, and the momentum seems to keep going in this direction. Over 1200 cases received first aid by participants with almost 100% rated as good management (airway and circulation management, limb fixation, and hemostasis). Another report by Nguyen concerned capacity improvement courses for lower-level injury in emergency facilities, which were conducted in 2016 with 220 medical staff [38]. The number of trainees with good first aid/trauma skills also increased markedly after completing these courses. The percentage of trainees who had good performance was over 80%. Therefore, the number of patients who needed to be transferred to a higher-level hospital subsequently decreased significantly.

Furthermore, Choi et al conducted a study on the outcome of a trauma education workshop in Vietnam [39]. The participants were highly satisfied with the quality of the workshop content (mean score 4.32, SD 0.62; measured by a 5-point Likert scale). The mean score of the teaching skill satisfaction and the perceived benefit from the workshop were both over 4 out of 5 points [39]. These types of evaluations have low face validity and reinforce the need for prospective studies looking at trainee knowledge and practice and patient clinical outcomes.

There are many short courses on first aid training in Vietnam annually. However, after completion, few courses reported course effectiveness, highlights, or learner feedback. The number of research or public papers from which data could be used was minimal. This limited number does not allow any further conclusions to be drawn from the data. Any analysis if done is usually a simple frequency analysis with no confidence scores. Because many courses have been run without being reported or updated, the exact or approximate numbers are difficult to ascertain.

## Discussion

### Principal Findings

This study aimed to review current trauma care training and describe the pattern of such training programs in Vietnam. We found that trauma care content was provided in both undergraduate and postgraduate training, as well as short-course training. However, the duration of such training was relatively short (under 5% of the curriculum has content related to trauma or first aid training). This result is similar to the report of inadequate training in trauma medicine of undergraduate students in the United Kingdom [40] or junior doctors in Australia [41]. Short-course training provided to medical staff who work in related fields might compensate for the deficiencies in undergraduate training.

### Long-term Solutions

As previously mentioned, due to rapid growth in the economy and therefore access to private transport, the number of road trauma cases has increased substantially. However, the ability of the Vietnamese health care system to adapt to this situation is questionable. Although, according to the World Health Organization, between 1997 and 2017, the numbers of medical education universities and graduated doctors have nearly doubled and trebled, respectively [42], an internal report of the MOH questioned the quality of the training and standards of these doctors. The problem is that there has not been a unique national examination for all medical universities that leads to a minimum standard among qualified doctors. To resolve this deficiency, the Vietnamese MOH has proposed some solutions. In 2017, the Vietnamese government issued a decree (75/2017/ND-CP) to facilitate the establishment of a “national medical exam” to validate and standardize the health care workforce [43]. Additionally, the Vietnamese government has issued a decree (109/2016/ND-CP) promulgating practice certificates to health care practitioners [44]. To obtain this certificate, health care workers must practice in a specialty for at least 18 months. After that, there is a requirement for them to attend at least 48 hours of training (CME); otherwise, their certification is canceled. The other initiative is a project named “Health Professionals Education and Training for Health System Reform (HPET),” which is being organized by the MOH and was launched in 2014. The objective of this project was to improve the quality of education and training of health personnel, health management, and capacity building of primary health care [45]. These efforts are considered a long-term method of changing trauma training and medical education.

Taking China as an example, the pattern of trauma and injuries has witnessed a similar increase but on a larger scale due to the huge population, while there are limited specialist trainings. The Chinese government has applied some solutions. First, the duration of trauma training for orthopedic residents has been increased from 6 to 16 months. Second, extra courses, such as “Arbeitsgemeinschaft fur osteosynthesfragen” (Association for the Study of Internal Fixation) (AO) Basic, AO Advanced, and AO Masters, have been offered and welcomed by orthopedic surgeons. Finally, some superior trainees have been sponsored by the Chinese Association of Orthopedics (CAOs) to attend fellowship training abroad [46]. Likewise, the Indian government endeavored to tackle new challenges in trauma care. Besides the raising of road safety awareness in the population, the short-course basic trauma life support (BLS) has been widely taught for both professionals and amateur bystanders. Additionally, the National Board of Examinations has recently begun registering courses in trauma care and the Academy of Traumatology (India) under the “National Trauma Management Course” (NTMC). These courses have accreditation from the International Association of Trauma Surgery and Intensive Care (IATSIC) and have attempted to standardized education in trauma life support skills [46,47].

All methods listed above are promising initiatives that hope to address the deeply rooted problem of fragmented and unvalidated trauma teaching. However, these strategies will take time to be implemented and show dividends. Thus, other more immediate actions are required in the interim. Moreover, because undergraduate programs have not favored specialty training, students are ineligible for clinical treatments including trauma management. Because of this circumstance, further education needs to be carried out as an interim endeavor.

### Short-term Educational Interventions

Since 2008, the MOH of Vietnam has come up with a solution that is Project 1816. This project has contributed to enhancing the qualifications of lower-level hospitals, including first aid and primary trauma care. This project deploys health professionals of highly specialized hospitals to support the hospitals of lower levels for at least 3-month secondments [11]. The support includes training and education on trauma knowledge and practice for both doctors and nurses. By doing this, health care workers at the grassroot level can learn from their actual issues at work and apply their obtained skills directly to their routine work [48].

Organizing more basic short courses, which contain a trauma component (such as “basic surgery” and “basic anesthesia”), is also an effective solution. The proportion of trauma care lessons in these specific courses is over 30% (Table 1), significantly higher than those in undergraduate or other postgraduate training programs. Moreover, the courses are especially suitable for medical staff working at the grassroots level, where they function as the “frontline station” for trauma victims, as the courses require only 6 to 11 months to complete.

**Table 1:** Basic short-course program.

Variable	Course^a^	
	Surgery	Anesthesia
Training duration (months)	6	11
Credits (theory/practice), n	33.5 (10.5/23)	37 (12/25)
Credits related to trauma, n (%)	10.5 (31.2)	12 (32.0)

^a^Qualified general doctors are eligible, and assessment is performed with an essay and a clinical base.

Another possible solution is to organize short courses in the CME format, in which the content focuses on trauma care, such as BLS and primary trauma care (PTC). These courses are designed for basic first aid applied in all health care centers with limited resources [49-51]. Peter et al showed improvements in both the knowledge (58%-77%) and confidence (68%-90%) scores of 1050 candidates after finishing a trauma management training program [49]. Another study conducted by Sadiq and Alwawi also reported that the mean knowledge score of participants had improved after primary trauma care courses (from 16/30 to 21/30 and 47.2% to 78.8%) [50,51]. However, these reports also have significant limitations. They failed to show any effect on patient outcome, which is considered as the final target of all medical education interventions. In Vietnam, the evidence of these courses is even weaker. Some reports showed that PTC had been well received by local participants without any further assessment [52-54]. For these reasons, research to evaluate the outcome of trauma training short courses, such as BLS and PTC, should be conducted in LMICs like Vietnam.

### Limitations

There are few papers on this topic, and they were mainly descriptive and lacked statistical methods. Because of this, we only conducted a narrative scoping review instead of a systematic review and meta-analysis. Hence, this article includes all the limitations of a scoping narrative method, including not formally assessing the quality of evidence and gathering information from a wide range of study designs and methods. In addition, we concentrated on the programs and curricula of HMU rather than all 27 medical schools in Vietnam. However, the differences among them were minimal, as they were all built according to a common MOH framework [55]. Moreover, due to limited resources, we could not include other types of medical education training, such as that for nursing and medical technicians.

### Conclusions

Medical training in Vietnam has not adapted to the emerging new condition of road trauma. To address this**,** the implementation of short courses, such as BLS and PTC, can be considered to compensate for the insufficient competency-related trauma care among health care workers while waiting for medical training reform.

## Appendix

Multimedia Appendix 1 Details of the search strategy.

## Abbreviations

BLS: basic trauma life support
CME: continuing medical education
HMU: Hanoi Medical University
LMIC: low- and middle-income country
MCQ: multiple-choice question
MOH: Ministry of Health
PTC: primary trauma care
RTI: road trauma injury
SL-I: specialized level I
SL-II: specialized level II
