# Supplementary material for: Trauma Care Training in Vietnam: Narrative Scoping Review
Source: JMIR Med Educ. 2022 Jan 24;8(1):e34369. doi: 10.2196/34369 (PMC8822435; doi:10.2196/34369)
Supplement: Multimedia Appendix 1 [file mededu_v8i1e34369_app1.docx]

**Multimedia Appendix 1.** Details of the search strategy.

Trauma care training in Vietnam: A narrative scoping review

Searching strategy in PubMed

| **Search**  **number** | **Query** | **Results** |
| --- | --- | --- |
| 19 | (Vietnam*) AND (((("health worker*") OR (physician*)) AND (((trauma) OR ("trauma care")) OR (injury))) AND (((((train*) OR (course)) OR (education)) OR (curriculum)) OR ((("continued medical education") OR ("continuing medical education")) OR (CME)))) | 20 |
| 18 | ((("health worker*") OR (physician*)) AND (((trauma) OR ("trauma care")) OR (injury))) AND (((((train*) OR (course)) OR (education)) OR (curriculum)) OR ((("continued medical education") OR ("continuing medical education")) OR (CME))) | 10,998 |
| 17 | ((((train*) OR (course)) OR (education)) OR (curriculum)) OR ((("continued medical education") OR ("continuing medical education")) OR (CME)) | 2,847,866 |
| 16 | (("continued medical education") OR ("continuing medical education")) OR (CME) | 35,200 |
| 15 | CME | 8,956 |
| 14 | "continuing medical education" | 28,429 |
| 13 | "continued medical education" | 152 |
| 12 | curriculum | 1,370,446 |
| 11 | education | 1,857,307 |
| 10 | course | 642,605 |
| 9 | train* | 688,254 |
| 8 | ((trauma) OR ("trauma care")) OR (injury) | 1,712,137 |
| 7 | injury | 1,573,753 |
| 6 | "trauma care" | 4,429 |
| 5 | trauma | 1,245,862 |
| 4 | ("health worker*") OR (physician*) | 713,761 |
| 3 | physician* | 693,411 |
| 2 | "health worker*" | 22,345 |
| 1 | Vietnam* | 30,448 |
